# In vivo protein turnover rates in varying oxygen tensions nominate MYBBP1A as a mediator of the hyperoxia response

**DOI:** 10.1126/sciadv.adj4884

**Published:** 2023-12-08

**Authors:** Xuewen Chen, Augustinus G. Haribowo, Alan H. Baik, Andrea Fossati, Erica Stevenson, Yiwen R. Chen, Nabora S. Reyes, Tien Peng, Michael A. Matthay, Michela Traglia, Alexander R. Pico, Daniel F. Jarosz, Abigail Buchwalter, Sina Ghaemmaghami, Danielle L. Swaney, Isha H. Jain

**Affiliations:** ^1^ Institute of Cardiovascular Disease, Gladstone Institutes, San Francisco, CA, USA.; ^2^ Department of Biochemistry and Biophysics, University of California San Francisco, San Francisco, CA, USA.; ^3^ Biomedical Sciences Graduate Program, University of California San Francisco, San Francisco, CA, USA.; ^4^ Department of Medicine, Division of Cardiology, University of California San Francisco, San Francisco, CA, USA.; ^5^ Quantitative Biosciences Institute (QBI), University of California San Francisco, San Francisco, CA, USA.; ^6^ Department of Cellular and Molecular Pharmacology, University of California San Francisco, San Francisco, CA, USA.; ^7^ Department of Chemical and Systems Biology, Stanford University, Stanford, CA, USA.; ^8^ Department of Medicine and Division of Pulmonary, Critical Care, Allergy and Sleep Medicine, University of California San Francisco, San Francisco, CA, USA.; ^9^ Bakar Aging Research Institute, University of California San Francisco, San Francisco, CA, USA.; ^10^ Cardiovascular Research Institute, University of California San Francisco, San Francisco, CA, USA.; ^11^ Departments of Medicine and Anesthesia, University of California San Francisco, San Francisco, CA, USA.; ^12^ Institute of Data Science and Biotechnology, Gladstone Institutes, San Francisco, CA, USA.; ^13^ Department of Developmental Biology, Stanford University, CA, USA.; ^14^ Department of Physiology, University of California San Francisco, San Francisco, CA, USA.; ^15^ Chan Zuckerberg Biohub, San Francisco, CA, USA.; ^16^ Mass Spectrometry Resource Laboratory, University of Rochester, Rochester, NY, USA.; ^17^ Department of Biology, University of Rochester, Rochester, NY, USA.

## Abstract

Oxygen deprivation and excess are both toxic. Thus, the body’s ability to adapt to varying oxygen tensions is critical for survival. While the hypoxia transcriptional response has been well studied, the post-translational effects of oxygen have been underexplored. In this study, we systematically investigate protein turnover rates in mouse heart, lung, and brain under different inhaled oxygen tensions. We find that the lung proteome is the most responsive to varying oxygen tensions. In particular, several extracellular matrix (ECM) proteins are stabilized in the lung under both hypoxia and hyperoxia. Furthermore, we show that complex 1 of the electron transport chain is destabilized in hyperoxia, in accordance with the exacerbation of associated disease models by hyperoxia and rescue by hypoxia. Moreover, we nominate MYBBP1A as a hyperoxia transcriptional regulator, particularly in the context of rRNA homeostasis. Overall, our study highlights the importance of varying oxygen tensions on protein turnover rates and identifies tissue-specific mediators of oxygen-dependent responses.

## Teaser

Ambient oxygen levels alter protein turnover rates in tissues, revealing mediators of the hyperoxia response.

## INTRODUCTION

Oxygen deprivation underlies countless pathological conditions including ischemic heart disease, hypoxic lung disease (e.g., emphysema), and stroke (*1*–*3*). Oxygen excess is also pathological in the context of hyperoxic lung injury, retinopathy of prematurity, and mitochondrial disorders (*4*–*7*). Variations in tissue oxygen levels trigger a multitude of adaptive and maladaptive signaling cascades. Most prior research in this space has focused on transcriptional responses mediated by the hypoxia-inducible factors (HIFs) (*3*, *8*, *9*). However, it is becoming increasingly clear that post-translational effects are also key regulators of the hypoxia response (*10*–*12*).

There are several known hypoxia adaptations that involve modulating protein turnover rates. Recently, researchers discovered an oxygen-sensing mechanism regulated by cysteamine dioxygenase (ADO) that is conserved from plants to mammals (*11*, *13*, *14*). In normoxic conditions, oxygen-dependent ADO modifies the N-terminal cysteines of target proteins, resulting in degradation via the N-end rule. In hypoxia, this reaction is inhibited, thereby stabilizing targets (*11*). Furthermore, hypoxia inhibits mammalian target of rapamycin (mTOR) signaling and activates the unfolded protein response (UPR) (*15*, *16*). These pathways decrease overall protein turnover rates in extreme hypoxia to conserve adenosine 5′-triphosphate (ATP) and alleviate endoplasmic reticulum stress (*16*).

On the other hand, oxygen-dependent changes in protein turnover can also be maladaptive. For example, we recently found that specific iron-sulfur (Fe-S)–containing protein complexes are destabilized and degraded in hyperoxia, leading to downstream biochemical defects (*17*). In particular, a substructure of electron transport chain (ETC) complex 1 (the matrix-facing arm that includes the N and Q modules) contains eight Fe-S clusters and is destabilized in hyperoxia. The resulting ETC dysfunction causes progressive hyperoxia, ultimately damaging additional Fe-S–containing proteins. Thus, the ETC is the “weakest link” in hyperoxia and is a primary cause of pathophysiology (*17*). In addition, post-translational disulfide bond formation is oxygen-dependent. Extreme hypoxia inhibits this reaction, causing protein misfolding and degradation (*18*).

Thus, there are clues in the literature that variations in oxygen levels can affect protein turnover rates in a manner that is adaptive or maladaptive. However, we lack a comprehensive investigation of protein turnover rates as a function of oxygen in vivo. More generally, most in vivo protein turnover studies have focused on baseline conditions rather than physiological responses to stress. It is also unknown how different organs cope with proteotoxic stress in hypoxia or hyperoxia. Lastly, while HIF is a well-studied transcriptional regulator of the hypoxia response, it is unclear whether there are analogous mediators of the hyperoxia response.

We set out to answer these questions using organism-level stable isotope-labeling proteomics (*19*). We focus on the lung, heart, and brain because these organs are particularly sensitive to pathologic conditions related to tissue hypoxia or hyperoxia. Our study highlights the tissue-specific responses to varying oxygen tensions and nominates MYB-binding protein 1a (MYBBP1A) as a transcriptional regulator of hyperoxia response. These findings are of particular relevance to states of pulmonary oxygen toxicity, including bronchopulmonary dysplasia in neonates and hyperoxic lung injury in adults.

## RESULTS

### Protein half-lives are determined by tissue-specific and protein-intrinsic features in normoxia

**Fig. 1.**
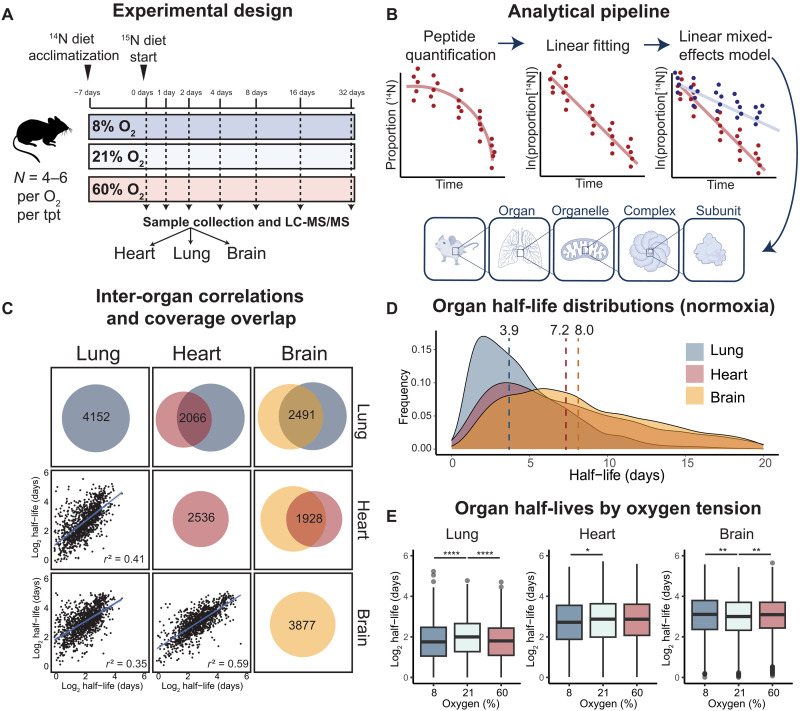
Pulsed SILAM reveals variations of protein half-lives across organs. (**A**) Schematic of the pulsed SILAM study design. Mice were acclimatized with the ^14^N algae diet in respective oxygen tensions for 7 days before the start of the experiment. At day 0, all mice were switched to the ^15^N-labeled algae diet to label all the newly synthesized proteins. Tissues were harvested at different time points following the start of the labeling. Lung, heart, and brain samples were analyzed using LC/MS-MS. (**B**) Data analysis schematics. The fraction of peptides made of ^14^N amino acids only was quantified and normalized at each time point to the values of 10 long-lived proteins (*22*). Following normalization, ^14^N fractions were fitted by linear mixed-effects models to estimate protein degradation rates for each protein in a given condition. To compare degradation rates between oxygen tensions, a linear mixed-effects model was applied where oxygen and time were set as fixed effects and peptides were random variables. (**C**) Study coverage, inter-organ overlap, and correlations. The circles represent the number of proteins in normoxia (21% O_2_) whose degradation rates were estimated in the study. The Venn diagrams represent the overlap of proteins measured in the study across organs. The scatterplots show the correlations of protein half-lives across organs, and the coefficients of determination (*r*^2^) are shown in the figures. (**D**) Density plots of protein half-lives and median half-lives in lung (3.9 days), heart (7.3 days), and brain (8.0 days) in normoxia across the three organs. (**E**) Protein half-lives across oxygen tensions. Statistical analysis was performed using the Kolmogorov-Smirnov test. *P < 0.05, **P < 0.01, and ****P < 0.0001.

To systematically study protein turnover rates in different oxygen tensions, we conducted stable isotope labeling of amino acids in mice (SILAM) (Fig. 1A). This approach relies on the assumption that the proteome is at steady state throughout the experiment. Thus, we first exposed wild-type mice to three different fractions of inspired oxygen (F_i_O_2_) for 1 week: chronic hypoxia (8% F_i_O_2_), normoxia (21% F_i_O_2_), or hyperoxia (60% F_i_O_2_). Throughout this pretreatment period, we placed mice on a control algae diet containing the standard ^14^N isotope. After 1 week, we switched the mice in different oxygen tensions to a ^15^N-containing algae diet and collected all major organs after 1, 2, 4, 8, 16, and 32 days. Tissue lysates were analyzed by data-independent acquisition (DIA) proteomics (*20*) to quantify the incorporation of labeled amino acids into the proteome of the lung, heart, and brain.

As previously shown, the incorporation of ^15^N into the proteome over time leads to broad MS1 spectra (*21*), which poses a challenge for accurate modeling and quantification of peptide turnover rates. Instead, it has been shown that protein turnover rates can be calculated by quantifying the decay of ^14^N peptides (due to ^15^N incorporation) (*19*). Therefore, to estimate protein degradation rates, we first quantified the decay of the ^14^N fraction over time. We then normalized the peptide abundance to that of 10 long-lived housekeeping proteins (table S1) that have previously been reported to have half-lives of greater than 2 months in mouse heart (Fig. 1B and fig. S1A) (*22*). We fitted these normalized data to a first-order kinetic model to estimate the protein degradation rate (*K*_d_) and applied a linear mixed-effects model to compare turnover rates across oxygen tensions (Fig. 1B and fig. S1A).

This allowed for the estimation of protein degradation rates and half-lives for 4152, 2536, and 3877 proteins in lung, heart, and brain, respectively (Fig. 1C, fig. S1B, and table S2). For greater than 50% of these proteins, the half-life was calculated using at least three detected peptides (fig. S1C). We observed a strong correlation of half-lives across biological replicates in each organ (Pearson correlation coefficient *r*: median = 0.95; range = 0.73 to 0.99), demonstrating the reproducibility of the dataset (fig. S1D).

We first examined normoxic tissues and observed marked differences in the distribution of protein half-lives across organs. The median half-life was 7.2 days in the heart, 8.0 days in the brain, and 3.9 days in the lung (Fig. 1D and fig. S1B). This may reflect more direct exposure of lung to the atmosphere. In addition, the lung has several cell types (e.g., epithelial cells, endothelial cells, etc.) that show greater overall turnover rates compared to terminally differentiated cell types in other organs (e.g., neurons and cardiomyocytes) (*23*–*25*).

Regardless of the overall distribution of protein half-lives, it is possible that the relative half-lives within the proteome are dictated at least partially by protein-intrinsic features. In line with this, we observed positive correlations of protein half-lives between normoxic brain, heart, and lung (*r*^2^ range: 0.35 to 0.59) (Fig. 1C), consistent with previous findings in primary cells and tissues (*21*, *26*). We further examined the intrinsic biophysical features of proteins, including peptide length, hydrophobicity, intrinsic disordered fraction, and charge patterns. We found that a larger intrinsic disordered fraction, less hydrophobicity, and larger fractions of negatively charged amino acids are associated with faster turnover rates in all three tissues (fig. S2A). Together, our data indicate that protein half-lives are dependent on intrinsic biophysical features of proteins but vary across organs.

### Tissue-specific features are the main determinant of protein half-lives in hypoxia and hyperoxia

**Fig. 2.**
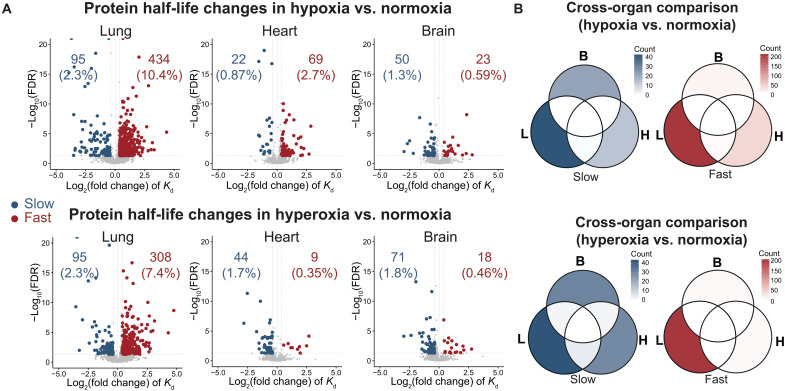
Protein turnover rates have different sensitivities to varying oxygen tensions in different organs. (**A**) Volcano plots of log_2_(fold changes) and −log_10_(FDR) of turnover rates (*K*_d_) for each protein calculated using a linear mixed-effects model. Oxygen and time were set as the fixed variables, and peptides were set as the random variables. Proteins with fold changes >1.3 and FDR < 0.05 are highlighted in blue (for proteins with slower turnover) or red (for faster turnover). Statistical analysis was performed using the linear mixed-effects model with Benjamini-Hochberg correction. (**B**) Venn diagrams of significantly changed proteins (fold change >1.3, FDR < 0.05). Proteins detected in all three organs were compared. The color gradients indicate the number of proteins that were significantly changed in lung (L), heart (H), or/and brain (B).

We next determined how variations in oxygen tension affect protein turnover rates across the three organs. We observed only modest changes in protein half-lives at the global level (Fig. 1E). Instead, most changes occurred in specific proteins. The lung had the greatest number of significantly affected proteins [fold changes > 1.3, false discovery rate (FDR) < 0.05]: 12.7% for hypoxia and 9.7% for hyperoxia (Fig. 2A). On the other hand, in the heart and brain, less than 4% of the proteins we monitored had significantly different turnover rates in hyperoxia or hypoxia (Fig. 2A).

There are two possible explanations for the marked effects on lung protein turnover rates: (i) The lung is directly exposed to environmental oxygen, so the local tissue partial pressure of oxygen (PO_2_) is most sensitive to changes in inhaled oxygen level, while for other tissues, oxygen levels are buffered by hemoglobin and partially normalized by physiological adaptations such as changes in hematocrit and vascular density; (ii) the overall cellular turnover rates in the lung may intrinsically be greater, requiring increased protein turnover (*27*). We observed minimal overlap between the significantly changed proteins across the three organs (Fig. 2B). In addition, we assessed the effects of protein-intrinsic properties. We found that the biophysical properties were not strong predictors of oxygen-dependent changes in protein turnover (fig. S2B). These results indicate that oxygen-dependent changes in protein turnover are highly variable across organs and are likely linked to tissue-specific physiological responses to hypoxia or hyperoxia.

### ECM proteins exhibit oxygen-dependent turnover rates in lung

**Fig. 3.**
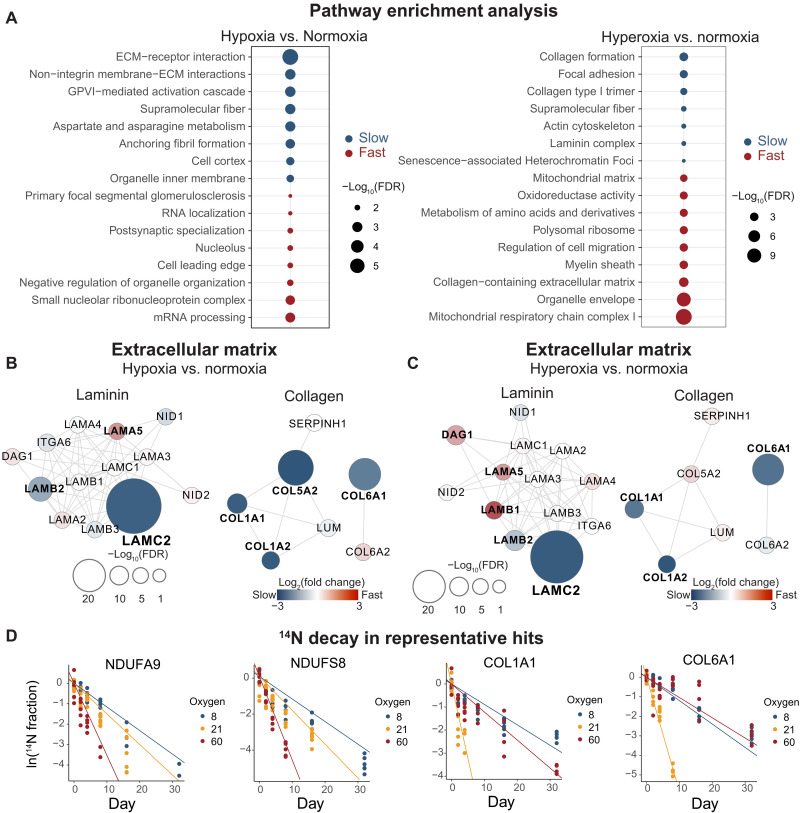
Extracellular matrix proteins exhibit oxygen-dependent turnover rates in lung tissue. (**A**) Enrichment analysis for proteins with significant changes in protein degradation rates (FDR < 0.05, fold change >1.3) in lung tissues using the STRING functional analysis method. The size of the dots represents −log_10_(FDR) for each term. (**B** and **C**) Protein-protein physical interaction network of extracellular matrix proteins in lung constructed using the STRING network function in Cytoscape (confidence score cutoff at 0.70). The plots show the laminin and collagen proteins and their first-degree neighbors in the network. Each node represents a protein that is measured in the lung, and edges represent the physical interactions. The color represents the fold change in protein degradation rates, and the size represents the significance level determined using the linear mixed-effects model. The proteins with FDR < 0.05 are bolded. (**D**) Representative scatterplots of ^14^N protein decay over time in the three oxygen tensions. Each dot represents the average abundance of all peptides in each subject. The fitted lines were calculated using the linear mixed-effects model, where the absolute numbers of the slopes correspond to the degradation rates.

We performed pathway enrichment analysis for proteins showing significant changes in turnover rates. In the lung, we found that proteins with slower turnover rates were enriched for extracellular matrix (ECM) remodeling pathways in both hypoxia and hyperoxia, including ECM-receptor interaction and collagen formation (Fig. 3A and table S3). In particular, collagen proteins (COL1A1, COL1A2, and COL6A1) and laminin proteins (LAMC2 and LAMB2) exhibit slower turnover rates in both hypoxia and hyperoxia than in normoxia (Fig. 3, B, C, and D, and table S3). Collagen and laminin are both critical components of the ECM, and have important roles in maintaining tissue integrity, providing structural support, and regulating cell behavior (*28*). There are different types of collagen and laminin proteins, with unique structural properties and functions. Of note, some laminin proteins, such as laminin subunit alpha 5 (LAMA5), showed higher turnover rates in hypoxia and hyperoxia than in normoxia (Fig. 3, B and C, and table S2), suggesting that different laminin subunits are regulated differently in response to oxygen changes. Our data reveal that these proteins are likely to be regulated post-translationally in varying oxygen tensions, potentially contributing to lung ECM remodeling. Notably, collagen is known to undergo oxygen-dependent prolyl hydroxylation, which affects protein stability (*29*).

Our data revealed distinct changes in protein dynamics in the heart and brain (fig. S3). In the heart, we observed increased degradation rates of proteins involved in ubiquinone (coenzyme Q) synthesis in hypoxia (fig. S3A, including COQ3 and COQ9; and table S3). In the brain, we found that proteins involved in receptor-mediated endocytosis, such as EFNB1 and EFNB2, a cell surface transmembrane ligand for Eph receptors, were stabilized under hyperoxic conditions (fig. S3D and table S3). Overall, our study provides insights into the tissue-specific responses to changes in oxygen that will serve as a broadly useful resource for the field.

### Oxygen-dependent effects on protein turnover rates vary across protein complex subunits

**Fig. 4.**
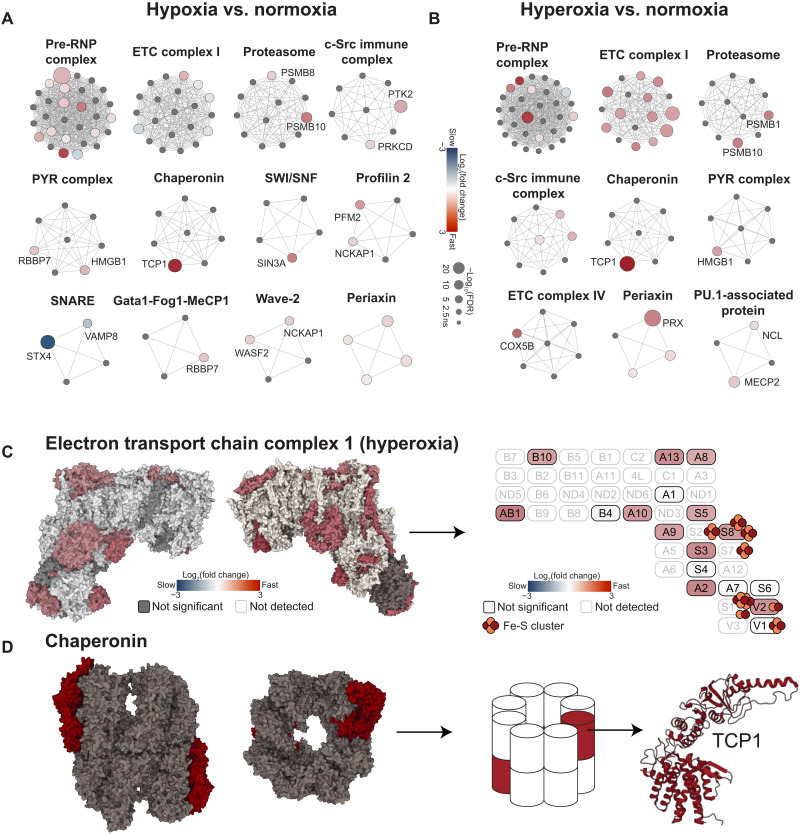
Effects of oxygen on protein complex turnover rates in lung tissue. (**A** and **B**) Network representation of protein complexes generated using Cytoscape. The protein complexes were retrieved from the CORUM database. Protein complexes with four or more subunits measured in the study are shown. The color gradient represents the differential protein degradation rates between oxygen tensions. Statistical analysis was performed using the linear mixed-effects model with Benjamini-Hochberg correction. Proteins with FDR < 0.05 are highlighted in blue (for proteins with slower turnover) or red (for faster turnover). The sizes of the nodes indicate the significance level. The edges represent the physical interactions among subunits in a protein complex. (**C**) Crystal structure and schematic of ETC complex 1 (*79*, *80*). The color gradient represents the fold changes of protein turnover rates in hyperoxia versus normoxia for the proteins that were significantly changed (FDR < 0.05) in their turnover rates. Oxygen labile Fe-S clusters are shown. (**D**) Crystal structure and schematic of chaperonin (*79*, *81*). TCP1, which had faster turnover rates in hypoxia and hyperoxia than in normoxia, is highlighted in red. Statistical analysis for all panels was performed using the linear mixed-effects model with Benjamini-Hochberg correction.

In our previous work, we found that hyperoxia destabilizes specific protein complexes containing Fe-S clusters (*17*). For example, the entire matrix-facing arm of mitochondrial ETC complex 1 was depleted in hyperoxia, suggesting complex-level effects, not just subunit-level changes (*17*). To determine whether this occurs more broadly, we searched all mammalian protein complexes using the CORUM database (*30*). This analysis confirmed that most ETC complex 1 subunits had shorter half-lives in hyperoxia in the lung (Figs. 3D and 4, B and C, and table S4). Among the 17 complex 1 subunits measured in this study, 11 subunits exhibited faster turnover rates in hyperoxia (2.1- to 3.7-fold) (Fig. 4, B and C). This was less evident in the brain and heart (table S2), likely because the inhaled hyperoxia is buffered by hemoglobin. Of note, tissue hyperoxia can also result from biochemical ETC deficiency [e.g., genetic mitochondrial disease (*4*)]. In this case, internal organs such as brain and heart will likely show more marked hyperoxia-dependent effects. A previous study demonstrated that subunits in the matrix-facing N-module of complex 1 are degraded at faster rates because of oxidative damage (*31*, *32*). In line with this, we found that the matrix-facing modules had significantly faster turnover rates than the membrane-bound module (fig. S4).

In contrast, in many other protein complexes, only one or two subunits are sensitive to varying oxygen levels (Fig. 4, A and B, and table S4). For example, the chaperonin complex contains eight subunits, but only t-complex 1 (TCP1) has faster turnover rates in both hypoxia and hyperoxia (Fig. 4D). This may be due to subunit-specific susceptibility to oxidation or protein-protein interactions. In the case of chaperonin, TCP1 is known to interact with von Hippel-Lindau tumor suppressor (VHL), an E3 ligase, to facilitate its folding (*33*). It is possible that there is increased oxygen-dependent interaction between TCP1 and VHL, resulting in increased ubiquitination and proteasomal degradation of TCP1. Alternatively, it is possible that the protein stability of specific proteins is affected before incorporation into the larger protein complex. In summary, oxygen-dependent changes in turnover rate do not always correlate across all proteins of a given complex.

### Hyperoxia-induced protein destabilization may exacerbate monogenic disorders

We previously reported that hyperoxia can worsen the disease progression of a mouse model of mitochondrial disease caused by genetic ETC defects, leading to respiratory failure and premature death (*4*, *34*). Moreover, hypoxia rescues disease and significantly improves survival, likely by acting on a fragile complex 1. In this study, we identified 434 proteins that were destabilized in hyperoxic lung. To investigate which other monogenic disorders may be exacerbated by hyperoxia, we crossed this list with the Online Mendelian Inheritance in Man (OMIM) database, a compendium of human monogenic disorders. Consistent with our previous findings, we identified many mitochondrial disease genes as hits (Table 1). In addition, we identified multiple disease genes that cause glycogen storage disease, spastic paraplegia, and Charcot-Marie-Tooth disease (Table 1). Patients with these conditions may be more sensitive to the use of supplemental oxygen and amenable to rescue by hypoxia exposure. Future work is needed to investigate the clinical and preclinical significance of these findings.

**Table 1.** Monogenic diseases that may be exacerbated by hyperoxia. Significantly destabilized proteins in hyperoxic lung with known monogenic disorders and phenotype MIM numbers.

Gene symbol	Gene name	Phenotypes and MIM numbers
GYG1	Glycogenin 1	Glycogen storage disease XV, 613507 (3), autosomal recessive; polyglucosan body myopathy 2, 616199 (3), autosomal recessive
GYS1	Glycogen synthase	Glycogen storage disease 0, muscle, 611556 (3), autosomal recessive
NDUFB10	NADH-ubiquinone oxidoreductase subunit B10	Mitochondrial complex I deficiency, nuclear type 35, 619003 (3), autosomal recessive
NDUFA13	NADH-ubiquinone oxidoreductase subunit A13	Mitochondrial complex I deficiency, nuclear type 28, 618249 (3), autosomal recessive
NDUFA2	NADH-ubiquinone oxidoreductase subunit A2	Mitochondrial complex I deficiency, nuclear type 13, 618235 (3), autosomal recessive
NDUFS8	NADH-ubiquinone oxidoreductase core subunit S8	Mitochondrial complex I deficiency, nuclear type 2, 618222 (3), autosomal recessive
NDUFA10	NADH-ubiquinone oxidoreductase subunit A10	Mitochondrial complex I deficiency, nuclear type 22, 618243 (3), autosomal recessive
NDUFA9	NADH-ubiquinone oxidoreductase subunit A9	Mitochondrial complex I deficiency, nuclear type 26, 618247 (3), autosomal recessive
NDUFA8	NADH-ubiquinone oxidoreductase subunit A8	Mitochondrial complex I deficiency, nuclear type 37, 619272 (3), autosomal recessive
NDUFV2	NADH-ubiquinone oxidoreductase core subunit V2	Mitochondrial complex I deficiency, nuclear type 7, 618229 (3), autosomal recessive
NDUFS3	NADH-ubiquinone oxidoreductase core subunit S3	Mitochondrial complex I deficiency, nuclear type 8, 618230 (3), autosomal recessive
B4GALNT1	β-1,4-N-acetylgalactosaminyltransferase 1	Spastic paraplegia 26, autosomal recessive, 609195 (3), autosomal recessive
CAPN1	Calpain, large polypeptide L1	Spastic paraplegia 76, autosomal recessive, 616907 (3), autosomal recessive
MTMR2	Myotubularin-related protein 2	Charcot-Marie-Tooth disease, type 4B1, 601382 (3), autosomal recessive
PRX	Periaxin	Charcot-Marie-Tooth disease, type 4F, 614895 (3), autosomal recessive; Dejerine-Sottas disease, 145900 (3), autosomal recessive, autosomal dominant

### Transcription cofactor MYBBP1A is stabilized in hyperoxic lung tissue

**Fig. 5.**
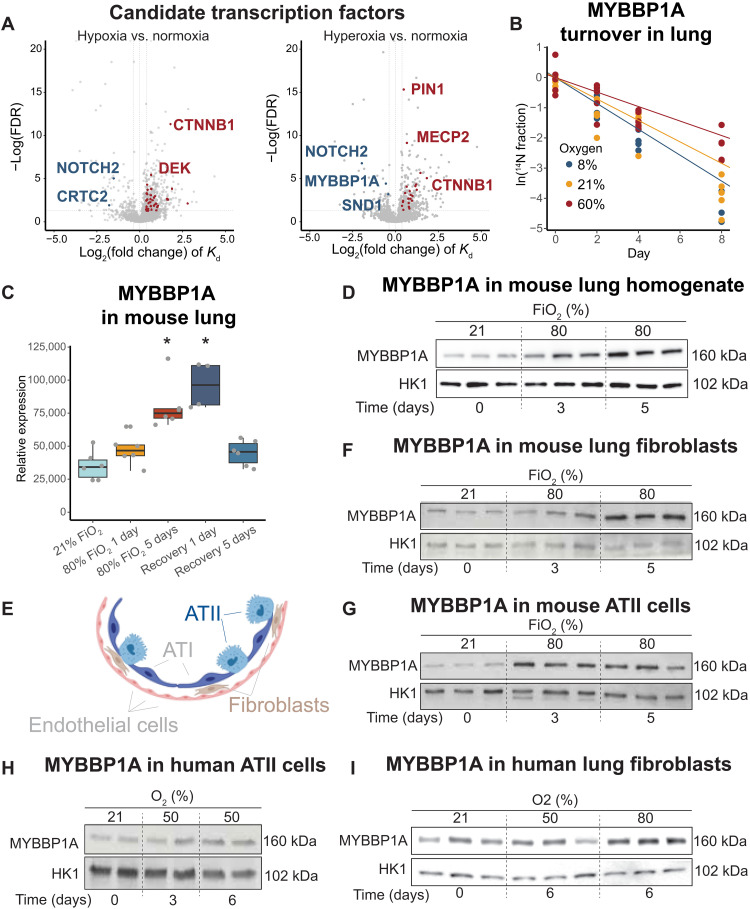
MYBBP1A is stabilized and accumulates in hyperoxic lung tissue. (**A**) Volcano plots of proteins with changes in protein turnover rates in the lung. Transcription factors and cofactors with significant changes are highlighted in blue (for proteins with slower turnover) or red (for faster turnover) in respective oxygen tensions. Statistical analysis was performed using the linear mixed-effects model with Benjamini-Hochberg correction. (**B**) ^14^N protein decay over time of MYBBP1A in lung tissues. Each dot represents the average abundance of all peptides in each subject. The corresponding fitted lines were calculated using the linear mixed-effects model, where the absolute numbers of the slopes correspond to the degradation rates. (**C**) Abundance proteomics data for MYBBP1A in mouse lung (*n* = 6 biological replicates per group) (*17*). Mice were exposed to 21 or 80% FiO_2_. For the recovery groups, mice were first exposed to 80% FiO_2_ for 5 days and brought back to 21% FiO_2_ for 1 or 5 days. Data were analyzed using unpaired *t* test following log transformation. *P value (with Benjamini-Hochberg correction) < 0.05. (**E**) Main cell types in lung tissues. Illustration made with Biorender. (**D**, **F**, and **G**) Western blots of MYBBP1A in mouse lung homogenates (D), isolated mouse lung fibroblasts (F), or mouse ATII cells (G) (*n* = 3 biological replicates per group). Mice were exposed to 21 or 80% FiO_2_ for up to 5 days. (**H** and **I**) Western blots of MYBBP1A in primary human ATII cells (H) and lung fibroblasts (I).

Hypoxia is known to stabilize HIF1α and HIF2α, which are critical transcription factors that drive hypoxia adaptations. We wondered whether there are specific transcriptional regulators that are similarly stabilized in hyperoxia, and may serve as mediators of a hyperoxia response. We searched for all transcription factors and cofactors in our dataset using the Animal TFDB 3.0 database (*35*). We found three such proteins with decreased turnover, indicating increased stability in lung tissue from hyperoxic mice: NOTCH2, MYBBP1A, and SND1 (Fig. 5, A and B, and fig. S5A). To investigate whether these transcription factors accumulate in hyperoxia, we reanalyzed an abundance proteomics dataset in lung homogenate from wild-type mice that were exposed to 21% O_2_ or 80% O_2_ for 5 days from our previous study (*17*). We also included samples from mice that were exposed to hyperoxia for 5 days and returned to normoxia for 1 or 5 days. We found that MYBBP1A protein levels in the lung progressively increased over the 5 days of exposure to hyperoxia (Fig. 5C). We validated this finding by Western blot of whole-lung homogenate from these same samples (Fig. 5D). The protein level remained elevated 1 day after the mice were transferred from 80 to 21% O_2_, as expected, because the protein’s half-life is ~2 days in normoxia. However, it normalized after 5 days of recovery in normoxia (Fig. 5C). These results demonstrate that MYBBP1A is stabilized under hyperoxia in lung tissue, resulting in its progressive accumulation. Moreover, this stabilization is reversible when the mice are returned to normoxic conditions. Next, to interrogate whether MYBBP1A is up-regulated in different lung cell types (Fig. 5E), we isolated lung fibroblasts and alveolar type II (ATII) cells from mice exposed to 21 or 80% O_2_. We demonstrated that MYBBP1A level was elevated in both cell types (Fig. 5, F and G). The timing of up-regulation varied: ATII cells responded after 3 days of hyperoxia, whereas fibroblasts showed an increase after 5 days (Fig. 5, F and G). This suggests varying sensitivities to hyperoxia among cell types. Furthermore, primary lung fibroblasts and ATII cells isolated from human patients also displayed increased MYBBP1A expression after being cultured in hyperoxic conditions (Fig. 5, H and I), highlighting the clinical relevance and evolutionary conservation of this response. These features make MYBBP1A a strong candidate for a regulator of the hyperoxia response. Therefore, we next focused on MYBBP1A and its downstream consequences.

### MYBBP1A is associated with rRNA processing in hyperoxic lung tissue

**Fig. 6.**
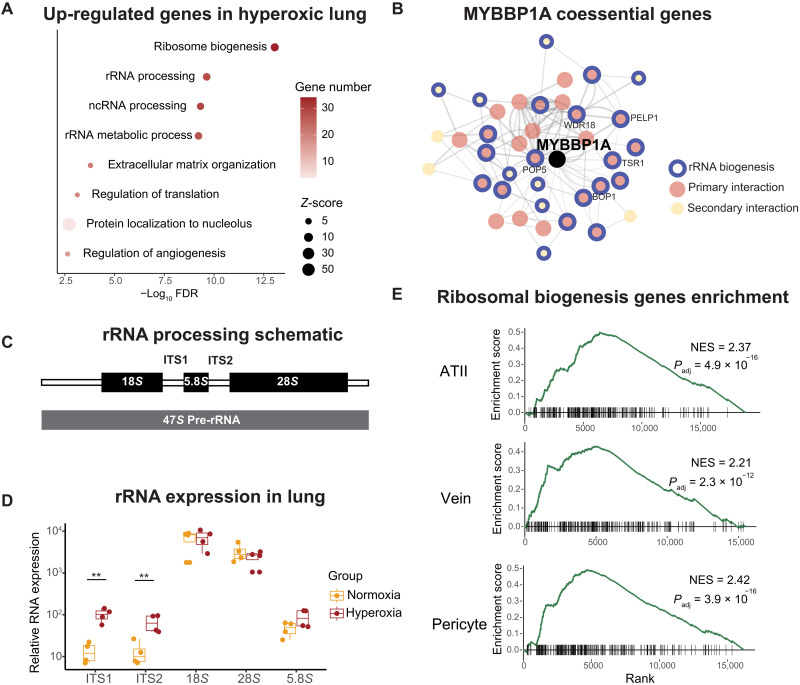
MYBBP1A up-regulation is associated with increased rRNA biogenesis in hyperoxia. (**A**) Top enriched pathways for up-regulated genes in hyperoxic lung [FDR < 0.05, log_2_ (fold change) > 1.5] using EnrichR. The color gradient represents the number of significantly up-regulated genes in each pathway. The Gene Ontology terms are ranked on the basis of the FDR values. (**B**) Coessentiality gene network of MYBBP1A in the DepMap database. The network was generated with the FIREWORKS web tool (*44*). Top 30 genes positively correlated with MYBBP1A are shown in red (primary), and the genes with the secondary interactions are shown in yellow. The thickness of the edges corresponds to Pearson correlations between two nodes (range = 0.21 to 0.65). Genes involved in rRNA biogenesis (GO:0042254) are circled with blue outlines. (**C**) Schematic of the rRNA precursor and mature ribosomal rRNA. (**D**) qRT-PCR of internal transcribed spacers (ITS1 and ITS2) of rRNA and mature rRNA, including 18*S*, 5.8*S*, and 28*S*. The RNA expression levels relative to *Hprt1* are shown. Each data point represents the average value of technical duplicates (*n* = 4 animals per group). Statistical analysis was performed using unpaired *t* test. **P < 0.01. (**E**) Genes in each cell type were ranked on the basis of the fold changes in the single-cell RNAseq data (*46*) (from the most up-regulated to the most down-regulated in hyperoxia). The genes involved in ribosome biogenesis (GO:0042254) were analyzed using the GSEA algorithm in each lung cell type. The cell types with the most significant enrichment are shown. NES, normalized enrichment score.

MYBBP1A has been studied in several cancer cell lines and *Drosophila*, where it is reported to be localized in the nucleolus (*36*–*40*). It has been proposed to have two primary roles: (i) as a cofactor of multiple transcription factors and (ii) as a regulator of ribosomal RNA (rRNA) processing/transcription (*41*). In the former case, MYBBP1A translocates to the nucleoplasm under stress conditions, where it represses the activity of certain transcription factors (e.g., PGC1α and MYB) and activates that of others (e.g., p53) (*40*–*42*). In the latter case, it is proposed to affect rRNA levels in the nucleolus (*36*, *37*). To better understand MYBBP1A’s function in an unbiased manner, we searched for genes that are coessential with MYBBP1A using the DepMap Genetic Dependency database. This resource identifies essential genes across hundreds of cancer cell lines using genome-wide CRISPR knockout screens (*43*). Genes that are most coessential across these cell lines are likely to be functionally related. We found that MYBBP1A is coessential with many other players in the rRNA biogenesis pathway (including WDR18, PELP1, POP5, BOP1, and TSR1), supporting the findings from previous literature (Fig. 6B and table S5).

To identify the potential effects of MYBBP1A in hyperoxia, we performed unbiased transcriptomics (using 3′ untranslated region mRNA sequencing, Quant-seq) to compare gene expression in normoxic versus hyperoxic mouse lung, matched to our previous abundance proteomics dataset. We found that genes that were up-regulated in lung tissue after exposure to 80% oxygen for 5 days were enriched in ribosome processing and rRNA biogenesis (Fig. 6A and tables S6 and S7). Moreover, we observed a moderate up-regulation of p53 targets in hyperoxia, including p21 (fig. S5B). Thus, it is possible that MYBBP1A up-regulation also promotes the p53-mediated transcriptional response. On the other hand, targets of MYB or PGC1⍺ were not significantly changed (fig. S5B). Therefore, we hypothesized that up-regulation of MYBBP1A in hyperoxia primarily affects rRNA processing in hyperoxia.

The rRNA precursor is a polycistronic sequence that is transcribed from rDNA and undergoes complex processing to produce three of the four mature rRNAs (18*S*, 5.8*S*, and 28*S*) in the nucleolus. The mature rRNAs are then assembled into the ribosome and are required for its function in protein translation (*45*). To investigate the effects of hyperoxia on rRNA biogenesis, we designed polymerase chain reaction (PCR) primers targeting the three mature rRNA and internally transcribed sequences (ITS) of the 47*S* rRNA precursor (Fig. 6C). We performed quantitative reverse transcription polymerase chain reaction (qRT-PCR) for pre-rRNA and mature RNA in mouse lung tissues exposed to 21% O_2_ or 80% O_2_ for 5 days. We found that lung tissues had a nearly 10-fold increase in pre-rRNA expression in hyperoxia compared to normoxia, whereas there were no significant changes in the expression of mature rRNAs (Fig. 6D). Thus, stabilization of MYBBP1A is associated with increased rRNA precursors in the hyperoxic lung.

Next, we set out to understand which lung cell types show MYBBP1A-mediated transcriptional changes. We analyzed a previously published single-cell RNA-sequencing (RNAseq) data for mouse lung exposed to 21 or 85% oxygen from birth to postnatal day 14 (*46*). We found that most endothelial, epithelial, and stromal cell types showed a significant increase in expression of genes involved in ribosomal biogenesis (fig. S5C), which is in concordance with our bulk Quant-seq data in hyperoxic lung tissue. In particular, ATII cells, pulmonary vein endothelial cells, and pericytes showed the most substantial fold changes (Fig. 6E). In addition, we crossed the single-cell RNAseq dataset with genes down-regulated in MYBBP1A knockout K562 cells (*47*). These genes are likely regulated by MYBBP1A-mediated transcription responses. In support of our overarching hypothesis, these genes are enriched in most of the lung cell types in hyperoxia, including ATII cells, pulmonary vein endothelial cells, and fibromyocytes (fig. S5D). Together, these analyses support the idea that MYBBP1A-mediated changes occur in a range of lung cell types during hyperoxia.

### The role of NAD+/NADH in mediating MYBBP1A stabilization in hyperoxia

**Fig. 7.**
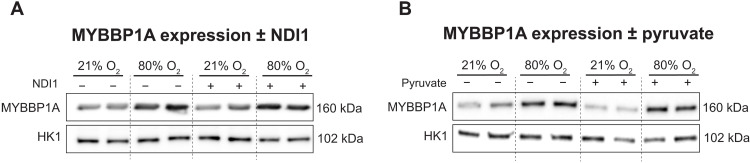
Effects of manipulating NADH recycling on MYBBP1A induction in hyperoxia. (**A** and **B**) Western blotting of human lung fibroblasts in 21% versus 80% oxygen for 6 days with NDI1 expression or pyruvate treatment. Biological duplicates are shown.

Destabilization of ETC complex 1 causes reductive stress resulting from decreased NADH recycling to NAD+ (*48*). To interrogate the effects of complex 1 loss on MYBBP1A stabilization, we expressed yeast NADH dehydrogenase (NDI1) protein in cells to restore the NAD+/NADH ratio in hyperoxia. Unlike mammalian complex 1, NDI1 does not contain Fe-S clusters but instead contains flavin adenine dinucleotide (FAD) as the electron carrier (*49*). We showed that expressing NDI1 in multiple cell lines increased the basal mitochondrial respiration rates and rotenone-resistant respiration rates in hyperoxia, demonstrating that NDI1 can at least partially complement the decrease in NADH-linked ETC respiration in hyperoxia (fig. S5, E and F). To test whether impaired NADH recycling underlies elevated MYBBP1A levels in hyperoxia, we transduced primary human lung fibroblasts with NDI1 and exposed them to different oxygen levels. NDI1 expression failed to reverse the up-regulation of MYBBP1A in hyperoxia (Fig. 7A). In addition, we treated the cells with sodium pyruvate to restore the NAD+/NADH ratio by increasing lactate dehydrogenase activity. The addition of pyruvate did not reverse the up-regulation of MYBBP1A (Fig. 7B). Therefore, impaired NADH recycling in hyperoxia is not sufficient to explain the up-regulation of MYBBP1A. Future work will be needed to decipher the signaling cascades linking hyperoxia to MYBBP1A accumulation and the downstream consequences.

## DISCUSSION

Oxygen plays a critical role in protein turnover rates via post-translational modifications. However, it is unclear how varying oxygen tensions affect protein turnover in vivo. In this study, we found that oxygen-dependent changes in protein turnover rates under hypoxic and hyperoxic conditions were highly tissue-specific. The lung, in particular, showed the most marked changes. We uncovered that oxygen-dependent changes of turnover rates in protein complexes were not always correlated. Furthermore, our results nominate MYBBP1A as a transcriptional regulator in hyperoxic lung, which may have important implications for lung pathophysiology.

Changes in protein turnover rates reveal tissue-specific proteome dynamics in response to hypoxia or hyperoxia. The proteins with significant changes in turnover rates showed minimal overlap across the three tissues. The top changing pathways were associated with tissue-specific responses to varying environmental oxygen levels. Our results demonstrated that in lung tissue, many ECM proteins showed changes in both hypoxia and hyperoxia, including collagen proteins and laminin proteins. ECM determines the architecture of lung via biochemical and biomechanical signals (*50*). Changes in the ECM composition and protein modifications occur in several chronic lung diseases (*50*). Previous literature has revealed that hypoxia and hyperoxia can have substantial effects on the ECM remodeling in the lung, via post-translational regulations. In hypoxia, collagen-modifying enzymes are up-regulated in a HIF-mediated manner, including prolyl 4-hydroxylase α-subunit isoform 1 and 2 (P4HA1/2), procollagen-lysine 2-oxyglutarate 5-dioxygenase 2 (PLOD2), and lysyl oxidase (LOX) (*51*, *52*). On the other hand, hyperoxia modulates matrix metalloproteinase (MMP) activities via transforming growth factor–β (TGFβ) signaling, resulting in increased stiffness of ECM (*53*, *54*). It is possible that these enzymes modify collagen and laminin proteins post-translationally, resulting in their stabilization in hypoxia or hyperoxia. This may contribute to increased collagen deposition and basement membrane disruption in lung ECM in varying oxygen tensions (*53*, *55*). Future studies should explore which post-translational modifications contribute to ECM protein stabilization in these contexts.

We found that protein turnover changes in protein complexes are not always correlated. Our previous study showed that most ETC complex 1 subunits are degraded in hyperoxia in cell lines and in mouse lung tissue (*17*). Consistent with this, we found that in the hyperoxic lung, most detected complex 1 subunits exhibited increased protein turnover rates. However, to our surprise, in other complexes, such as the chaperonin and proteasome, only specific proteins showed significant changes. The labile subunits in these complexes may be regulated by interactions with other proteins or post-translational modifications (*33*, *56*).

In addition, our findings shed light on potential therapeutic targets for diseases related to chronic hypoxia or hyperoxia, including chronic lung disease, hyperoxic lung injury, ischemic heart disease, and mitochondrial disease. We found that ETC complex 1 subunits were destabilized in hyperoxic lung, which is in accordance with our previous studies demonstrating that hyperoxia exacerbates and hypoxia rescues mitochondrial complex 1 disease. In addition, we nominated three other monogenic diseases that may be worsened by tissue hyperoxia, including glycogen storage disease, spastic paraplegia, and Charcot-Marie-Tooth disease.

Furthermore, we nominated MYBBP1A, a transcriptional regulator, as a dynamic mediator of the hyperoxia response. We found that MYBBP1A was stabilized in hyperoxic lung tissue, resulting in its accumulation in multiple lung cell types. Notably, the protein level returned to baseline levels after return to normoxia. MYBBP1A is predominantly localized in the nucleolus, where it regulates rRNA biogenesis and plays a critical role in the assembly of ribosomes (*37*). Our data showed an up-regulation of rRNA biogenesis genes in hyperoxic lung homogenates, as well as several key lung cell types, suggesting that MYBBP1A may play a role in rRNA regulation in hyperoxia. It has been shown that knockdown of *Mybbp1a* in *Drosophila* affects pre-rRNA and mature rRNA levels and causes developmental defects (*36*). In contrast, *Mybbp1a* overexpression suppresses RNA polymerase I activities (*37*), indicating its complex regulatory role in rRNA biogenesis. Therefore, we investigated the levels of rRNA in varying oxygen tensions. We found that in hyperoxic lung tissues, the pre-rRNA level was increased. This increase may lead to MYBBP1A retention and stabilization in the nucleolus. Overall, our findings shed light on the critical role of MYBPP1A in rRNA biogenesis in hyperoxic lung. Future studies will causally delineate the complex role of MYBBP1A in hyperoxic transcriptional regulation by knocking out the gene in different lung cell types.

Future studies will also investigate the mechanisms underlying MYBBP1A stabilization in hyperoxia and test whether this is adaptive or maladaptive. We demonstrated that impaired NADH oxidation due to impaired complex 1 activity is not required for hyperoxic stabilization of MYBBP1A. Follow-up studies will investigate the role of several known regulators of MYBBP1A turnover in hyperoxia signaling—for example, PREP1 and USP29 have been shown to stabilize MYBBP1A, whereas VHL degrades it (*57*–*59*).

Limitations of our study include the fact that it spans from 1 day to 32 days, which only allows us to estimate protein half-lives that fall into that range. If proteins have half-lives shorter than 12 hours or longer than 32 days, we are not able to accurately estimate half-lives. As a result, we may miss some proteins with oxygen-dependent half-lives. The model we use is based on the assumption that protein abundance is constant over the course of the study. We have previously shown that acute hypoxia causes marked changes in behavior and tissue oxygenation, but these functions are normalized after ~1 week (*60*). Thus, we acclimatized the mice in respective oxygen tensions for 1 week before the start of the isotope labeling. However, we cannot rule out that the expressions of some proteins fluctuate (e.g., circadian proteins) throughout our experiment. We showed that the stabilization of MYBBP1A is associated with hyperoxia responses including alterations in rRNA biogenesis and increased expressions of p53 targets. However, future studies are needed to causally prove this.

In summary, our study provides a comprehensive analysis of in vivo protein turnover rates in varying oxygen tensions. This is relevant for a broad range of clinical conditions including chronic lung disease, hyperoxic lung injury, ischemic heart disease, and mitochondrial disease. This work sheds light on hypoxia and hyperoxia-labile protein complexes and subunits that may be relevant for several monogenic disorders. Moreover, it serves as a valuable resource for future studies of hypoxia and hyperoxia responses that are mediated by changes in protein stability. As a key example, we nominate MYBBP1A as a regulator of the hyperoxia response that is associated with changes in rRNA homeostasis. By discovering mediators of oxygen-dependent responses, we can identify therapeutic targets for states of mismatched oxygen supply and demand.

## MATERIALS AND METHODS

### Animal model

C57BL/6J (IMSR_JAX: 000664) male mice (7-week-old) were purchased from the Jackson Laboratory. Upon delivery, the mice were housed in the University of California, San Francisco (UCSF) animal facility. Different oxygen levels (8, 60, or 80% O_2_) were created by mixing N_2_ (Airgas), O_2_ (Airgas, Praxair), and room air carefully controlled by gas regulators. The O_2_ and CO_2_ levels were continuously monitored with wireless sensors and checked daily. To prevent CO_2_ buildup in the chambers, soda lime (Fisher Scientific, A1935236) was placed inside the chambers to absorb CO_2_. All experiments were performed on C57BL/6J mice between the ages of 8 and 12 weeks. All animal studies were approved by the Institutional Animal Care and Use Committee Program at UCSF.

### Cell models

K562 [American Type Culture Collection (ATCC), CCL-243] and A549 (ATCC, CCL-185) cells were purchased from ATCC and were maintained in Dulbecco’s modified Eagle’s medium (DMEM; Gibco/Life Technologies, 11995073) supplemented with 10% fetal bovine serum (FBS; Corning/Fisher Scientific, MT35015CV) and 1% penicillin-streptomycin (Fisher Scientific, 15140122). Human lung fibroblasts (HLFs; Sigma-Aldrich Inc., 506-05A) were maintained in DMEM/F-12 (Thermo Fisher Scientific, 11330032) supplemented with 10% FBS and 1% penicillin-streptomycin. Human ATII epithelial cells were isolated from human lungs declined for transplantation by the Northern California Transplant Donor Network as previously described (*61*). Cells were plated at 1 × 10^6^ cells per well on collagen I–coated Transwell plates (Corning, CLS3495, Sigma-Aldrich) in an air-liquid interface. Cells were maintained in 50% DMEM high-glucose/50% F-12 mix supplemented with 1% penicillin-streptomycin, 1% fungisome, and 0.1% gentamicin. Mycoplasma tests were performed quarterly on all cell lines. All cells were maintained in cell culture incubators (37°C, 5% CO_2_). The oxygen tension in the hyperoxia cell culture incubator was created by mixing compressed air and 100% O_2_ (Praxair).

### Isotope labeling of mice and tissue collection

C57BL/6J male mice (four to six mice per time point) were fed on ^14^N and ^15^N mouse chow (obtained from Silantes). Animals were first acclimatized in respective oxygen tension with ^14^N (normisotopic) food for 1 week. Mice in the hypoxia group were first acclimatized in 11% O_2_ for 1 week before transferring to 8% O_2_. The animals were then transitioned to ^15^N chow throughout the labeling period (for 1, 2, 4, 8, 16, and 32 days). Mice were sacrificed by isoflurane inhalation. The animals were perfused via the left ventricle with ice-cold PBS for 2 min. Heart, lung, and brain tissues were flash-frozen in liquid nitrogen.

### Proteomics sample preparation

Tissues were processed using the SPEED (sample preparation by easy extraction and digestion) preparation method (*62*). Briefly, 40 μl of trifluoroacetic acid (TFA) was added and incubated for 3 min at 70°C. TFA was neutralized by adding 10 volumes of neutralization buffer (2 M Trizma base in H_2_O). Cysteines were reduced and alkylated with 5 mM Tris(2-carboxyethyl)phosphine (TCEP) and 10 mM chloroacetamide, and the samples were incubated at 95°C for 5 min. Protein quantification was performed via the Bradford assay, and all samples were adjusted at 100 μg. The resulting volumes were diluted 1:1, and 1 μg of trypsin was added. Proteins were digested overnight at 37°C on a thermo-shaker (600 rpm). Peptides were desalted using a 96-well format C18 plate (Nest group) following the manufacturer’s instruction and dried under vacuum.

### Mass spectrometry acquisition

The lung samples were resuspended in buffer A [0.1% formic acid (FA)], and approximately 200 ng was analyzed by DIA-PASEF (parallel accumulation-serial fragmentation combined with data-independent aquisition) (*63*) on a Bruker TimsTof Pro 2 interfaced with an Ultimate3000 UHPLC. The peptides were separated on a PepSep column (15 cm length, 150 μm inner diameter) using a 38-min gradient at 0.5 μl/min. Following loading, the peptides were eluted with a 5 to 30% buffer B [0.1% FA in acetonitrile (ACN)] in 20 min. The column was then washed for 5 min at 90% and high flow (1 μl/min) and reequilibrated with 5% ACN for the next run. The peptides were sprayed on a glass capillary kept at 1700 V and 200°C. The mass spectrometer was operated in a positive mode using the DIA-PASEF acquisition (*63*). Briefly, four PASEF scans (0.85 1/K0 to 1.30 1/K0) were acquired and divided each precursor range into 24 windows of 32 Da [500.7502 to 966.67502 mass/charge ratio (m/z)] overlapping 1 Th.

The heart and brain samples (approximately 500 ng) were directly loaded on an Evosep C18 tip and separated using the Evosep One using the 60 spd method (Evosep, Odense, Denmark). Peptides were eluted and ionized using a Bruker Captive Spray emitter. A Bruker TimsTof Pro 2 mass spectrometer running in DIA-PASEF mode (*63*) was used for acquisition. The acquisition scheme used for dia-PASEF consisted of 6 × 3 50 *m*/*z* windows per PASEF scan.

### Lung cell isolation

C57BL/6J male mice were sacrificed by isoflurane inhalation, soaked in 70% ethanol for 1 min, and then perfused with PBS. The heart tissues were removed, and the trachea was tied with a loose knot. The lungs were insufflated with dispase, collagenase, and deoxyribonuclease I. Next, the lungs were removed and immersed in dispase in 37°C for 45 min on a rocker to digest the lungs. To prepare for sorting, the suspension was filtered through a 70-μm filter and rinsed with sorting buffer (DMEM/F12, no phenol red, with 2% FBS, and 1% penicillin-streptomycin). The cells were spun down at 550*g* at 4°C for 5 min and resuspended in red blood lysis buffer. The cells were filtered through a 40-μm filter, rinsed, and spun down at 550*g* at 4°C for 5 min. Cells were mixed with rat serum at 4°C for 10 min to block nonspecific binding. Cells were incubated in biotin-conjugated primary antibodies (CD45, CD31, CD46, and Ter^199^) for 30 min at 4°C and Streptavidin beads at room temperature (RT) for 3 min to remove immune and endothelial cells. Cells were then stained with Epcam, integrin B4, and major histocompatibility complex class II (MHC-II) for 30 min at 4°C. Live AT2 cells (lin^−^ Epcam^+^ B4^−^ MHC-II^+^) and line fibroblast (lin^−^ Epcam^−^) were sorted with a flow cytometer (*64*, *65*). See table S8 for the catalog numbers and dilutions of the antibodies. Biological triplicates were analyzed in the study, and two mice were pooled for each replicate. Cells were pelleted and stored at −80°C.

### Western blotting

C57BL/6J mice were exposed to room air or 80% O_2_ (*n* = 3 animals per group). Tissues were perfused with chilled Dulbecco’s phosphate-buffered saline (DPBS; Corning). Lung tissues were collected and flash-frozen in liquid nitrogen. Lung tissues were homogenized using the Qiagen Tissue Lyser II (30 Hz, 1 min) in radioimmunoprecipitation assay (RIPA) buffer (Thermo Fisher Scientific, PI89901) with cOmplete Protease Inhibitor Cocktail (Roche), followed by sonication. Cell pellets were lysed in RIPA buffer with cOmplete Protease Inhibitor Cocktail. Protein concentrations were determined using the Rapid Gold BCA Protein Assay Kit (Thermo Fisher Scientific, A53225). Equal amounts of protein were mixed with 6× Laemmli SDS sample buffer (Fisher Scientific, AAJ61337AD), and samples were boiled at 95°C for 5 min. Protein lysates were run on SDS–polyacrylamide gel electrophoresis gels (Mini-PROTEAN TGX Precast Protein Gels) at 200 V and were transferred onto polyvinylidene difluoride membranes. Membranes were blocked with 3% nonfat milk (Genesee Scientific Corporation, 20-241) in Tris-buffered saline, 0.1% Tween 20 (TBST) (Fisher Scientific, 28360) for 1 hour at RT on a rocker. The membranes were probed with primary antibodies overnight at 4°C (anti-MYBBP1A, Proteintech, 14524-1-AP, 1:1000; anti-HK1, Cell Signaling Technology, 2024, 1:1000). The corresponding secondary antibodies were applied to the membranes for 1 hour at RT (anti-rabbit horseradish peroxidase, VWR 95017-556; 1:5000). Bands were visualized using enhanced chemiluminescence (Fisher Scientific, PI32106) on the Western Blot Imaging System (Azure Biosystems) or x-ray films (GE Healthcare). Uncropped blot images have been deposited to Mendeley Data (doi: 10.17632/jx73jbnj3g.1).

### RNA extraction

Mouse lung tissues were perfused with cold DPBS and flash-frozen in liquid nitrogen. Tissues were homogenized in TRIzol reagent (Thermo Fisher Scientific, 15596026) using the Qiagen Tissue Lyser II (30 Hz, 2 min). RNA extraction was performed according to the manufacturer’s instructions. RNA purity and integrity were determined using NanoDrop One (Thermo Fisher Scientific) and Bioanalyzer (Agilent), respectively.

### Quant-seq 3′ mRNA-seq

The Quant-seq library preparation was carried out using the Lexogen Quant-seq 3′ mRNA-Seq V2 Library Prep Kit with unique dual index (UDI; #191.96). Equal amounts of RNA (500 ng) from samples (*n* = 7 for normoxia and *n* = 6 for hyperoxia) were used for the preparation, according to the manufacturer’s guide. Following the PCR amplification, the complementary DNA (cDNA) concentrations were examined using a Qubit double-stranded DNA High Sensitivity and Broad Range Assay Kit (Invitrogen Q32851) on a Qubit 4 fluorometer (Thermo Fisher Scientific). The average library sizes were determined using TapeStation 4200 (Agilent). Next, equal amounts of cDNA with UDIs were pooled and sequenced on an Illumina’s NovaSeq 6000 System in the UCSF Center for Advanced Technology (CAT).

### Quantitative reverse transcription polymerase chain reaction

RNA was reverse-transcribed using the QuantiTect Reverse Transcription Kit (Qiagen, 205311) according to the manufacturer’s instructions. The qPCR reactions were performed using qPCR primers targeting different loci of the rRNA (Fig. 6C and table S8) (*66*) and Maxima SYBR Green/ROX qPCR Master Mix (Thermo Fisher Scientific, K02222) on a QuantStudio 5 real-time PCR machine (Applied Biosystems). *Hprt1* was used as the housekeeping gene. RNA expressions relative to *Hprt1* were calculated using the delta Ct method. The experiments were performed in four biological replicates and technical duplicates. Unpaired *t* test was used for statistical analysis.

### Generation of NDI1-expressing cell lines

Retroviruses were generated by transfecting PMXS-NDI1 (Addgene, 72876) plasmid or empty vector in human embryonic kidney 293T cells (ATCC, CRL-3216) with the packaging plasmids pVSVg (Addgene, 8454) and psPAX2 (Addgene, 12260). K562, A549, and HLF cells were transduced with NDI1 or empty vector virus by spinfection as previously described (*34*). Cells were selected with blasticidin (Gibco, A1113903) at 1 μg/ml for 4 days.

### Mitochondrial respiration assay

K562 cells were pretreated in 21 or 50% O_2_ for 4 days. Cell-Tak (25 μg/ml) was used for acute adhesion on the Seahorse XFe96 Cell Culture Plates. On the assay day, 4.5 × 10^4^ cells were plated per well. The plates were centrifuged at 600*g* for 5 min. A549 cells were pretreated in 21 or 50% O_2_ for 3 days. An equal number of cells (1.5 × 10^4^) were plated on the Seahorse plate and incubated at respective oxygen tensions for an additional day. The assay medium was composed of DMEM powder (Sigma-Aldrich, D5030), 5 mM Hepes, 30 mM NaCl, 8 mM glucose, 2 mM pyruvate, and 2 mM glutamine (pH 7.4). Port A of the Seahorse XFe96 Sensor Cartridges was loaded with rotenone (final concentration = 500 nM). Port B was loaded with antimycin A (final concentration = 1 μM). The experiment was performed in three biological replicates and five technical replicates. Each well was measured five times at baseline and after each injection. The basal oxygen consumption rate (OCR) was determined by subtracting the average OCR by antimycin A injection from the average OCR in baseline. The rotenone-resistant OCR was determined by subtracting the average OCR after antimycin A injection from the average OCR after rotenone injection.

### MS data processing

The DIA-PASEF data were searched with DIA-NN v1.7.1 (*67*) using a library-centric approach. Identified spectra with MS1 precursors within 10 parts per million (ppm) and MS2 precursor within 15 ppm were selected, and a second library was generated (double-pass mode). Quantification was set to robust (high accuracy) while signal was renormalized as function of the spray stability (RT dependent). Protein inference was disabled, and library generation was set to smart profiling. The transition level data were filtered at 1% library Q-value, and transitions were summed into precursor MS2 abundances, and precursors were averaged to a single-peptide abundance.

### Degradation rate calculation and statistical analysis

The degradation rate (*K*_d_) is determined using a first-order kinetic model (*19*), under the assumption that the protein abundance remains constant and that protein synthesis is a zero-order process. The fraction unlabeled peptides (without ^15^N) can be modeled as a first-order kinetic model over the course the labeling time

$$\mathrm{Fraction}\;{}^{14}N\;(t)=e^{-k_{d}\times t}$$

The unlabeled peptide abundance was first centered using previously reported long-living proteins (*22*) under the assumption of fixed abundance between different conditions and labeling time. The normalized abundance was divided by the abundance at time 0 of the labeling to calculate the fraction of unlabeled peptides at each time point. Next, a preliminary fitting was performed to filter peptides with good linear correlations using the Ordinary Least Square (OLS) function from the Python (v3.11) statsmodels package (v0.13.5). Each peptide for a given condition was fit into the first-order kinetic model using the ordinary linear model, and the goodness of fit was assessed by *r*^2^. For the subsequent analysis, only peptides with *r*^2^ > 0.65 that are detected in more than five samples in a given condition were used for the fitting.

To calculate the *K*_d_ for each protein, a linear mixed-effects model was applied using the mixedlm function from the python (v3.11) statsmodels package (v0.13.5). For each oxygen condition and each tissue, time was set as the fixed variable, and to account for the variability of different peptides for a given protein, the peptides were set as the random variable. *K*_d_ values with *P* values less than 0.20 were reported. Protein half-lives (*t*_1/2_) were calculated on the basis of

$$t_{1/2}=\frac{\ln(2)}{K_{d}}$$

To compare *K*_d_ between oxygen tensions, a linear mixed-effects model using mixedlm with oxygen, time, and oxygen:time as fixed effects, and peptides as random variables was applied. The *P* values of the interaction between oxygen and time (oxygen:time) indicated the differences in the slopes, which assessed the differences of the degradation rates. Adjusted *P* values were calculated using the Benjamini-Hochberg correction.

### Quant-seq sequence alignment and data processing

The data alignment and analysis were carried out using the BioJupies automated analysis pipeline (*68*). The genes were aligned and the expressions were quantified using the kallisto pseudoaligner (v0.46.1) (*69*). The data were normalized using the count per million method. The statistics analysis was performed using limma (v3.54.2) (*70*).

### Biophysical feature analysis

The UniProt mouse canonical protein sequences (downloaded on 21 August 2022) were used as input to calculate various protein sequence features as described below. “Disordered fraction” was computed on the basis of the fraction of total residues with a score above 0.5 using the DISOPRED3 method (*71*). “Longest disordered stretch” refers to the length of the longest disordered domain (defined as a continuous stretch of sequence with each amino acid having a DISOPRED3 score above 0.5). “Disorder promoting” was computed on the basis of the fraction of residues that is predicted to be disorder promoting using the TOP-IDP–scale method (*72*). “Fraction expanding” is defined by the fraction of residues that contribute to chain expansion (E/D/R/K/P) calculated using CIDER (*73*). “Delta” score is a parameter for protein charge patterning calculated using the localCIDER algorithm (*73*). “Fraction charged AA”, “Fraction AA^−^”, and “Fraction AA^+^” refer to the fraction of charged residues (H, K, R, D, and E), the fraction of negatively charged residues (D and E), and the fraction of positively charged residues (H, K, and R), respectively. “Hydropathy” is the mean hydropathy as calculated from a skewed Kyte-Doolittle hydrophobicity scale (*74*). “Length” refers to the protein amino acid length.

For enrichment analysis at baseline, the proteins are ranked on the basis of the degradation rates. The top 5% and bottom 5% of proteins from each organ were used to compare with the background distribution (all other proteins). For the analysis for the oxygen-dependent changes, the proteins were ranked by *t*-statistics calculated using the linear mixed-effects model described above. The top 2% and bottom 2% of proteins from each organ were used to compare with the background distribution (all other proteins). The enrichment scores were calculated using the Kolmogorov-Smirnov test.

### Pathway analysis

For the pulsed-SILAM dataset, proteins with fold changes >1.3 and FDR < 0.05 in protein turnover rates between different oxygen tensions were imported into Cytoscape (3.9.1) (*75*). The enrichment analysis was performed using the STRING Functional Enrichment in the Cytoscape (confidence score cutoff: 0.70). Pathways with fewer than 1000 proteins were selected and were ranked on the basis of *P* values.

For the Quant-seq dataset, RNA transcripts with absolute number of log_2_(fold change) > 1.5 and FDR < 0.05 were selected, and the enrichment analysis was performed using EnrichR, and the genes were mapped against the mouse Gene Ontology database (*76*).

The transcription factor target lists were curated from the Cistrome database (*77*). All the datasets that were generated in mice and passed the peak quality control were merged. The binding targets that were found in at least 50% of all datasets were selected for the analysis. The enrichment scores were calculated using the Gene Set Enrichment Analysis (GSEA) (*78*).

For the single-cell RNAseq data (*46*), genes were ranked by fold changes for each cell type. The pathway enrichment was performed using GSEA.

### Protein complex analysis

The protein complex data were retrieved from CORUM (on 3 September 2018) (*30*). The data were imported to Cytoscape (3.9.1) to create the network representations.
