# Supplementary material for: In vivo protein turnover rates in varying oxygen tensions nominate MYBBP1A as a mediator of the hyperoxia response
Source: Sci Adv. 2023 Dec 8;9(49):eadj4884. doi: 10.1126/sciadv.adj4884 (PMC10708181; doi:10.1126/sciadv.adj4884)
Supplement: Supplementary file 1 — Figs. S1 to S5 Legends for tables S1 to S8 [file sciadv.adj4884_sm.pdf]

Science Advances
AAAS

Supplementary Materials for

In vivo protein turnover rates in varying oxygen tensions nominate MYBBP1A as a mediator of the hyperoxia response

Xuewen Chen, *et al.*

Corresponding author: Isha H. Jain,isha.jain@gladstone.ucsf.edu

Sci. Adv. **9**, eadj4884 (2023)
DOI: 10.1126/sciadv.adj4884

The PDF file includes:

Figs. S1 to S5
Legends for tables S1 to S8

Other Supplementary Material for this manuscript includes the following:

Tables S1 to S8

SUPPLEMENTAL FIGURES

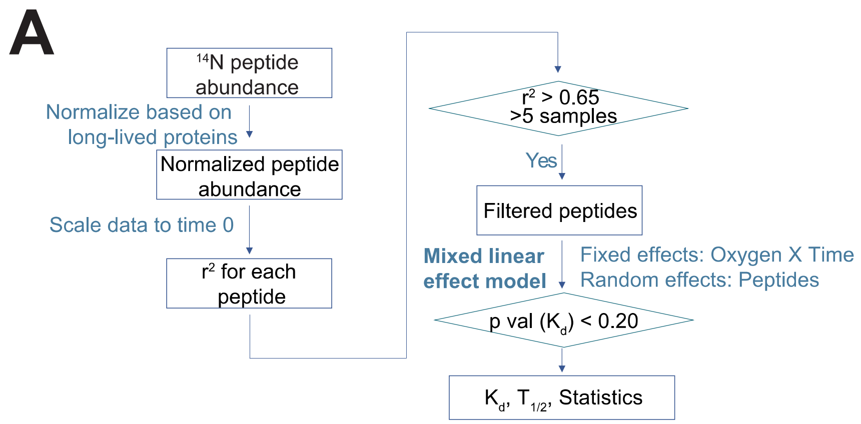

B

	Oxygen	Total Peptide Number	Filtered Peptide Number	Protein Number	Median Half-lives (days)
Lung	8%	31457	14848	3628	3.21
	21%	31805	10338	3190	3.85
	60%	31606	11108	3342	3.29
Heart	8%	26393	8901	2128	6.50
	21%	26563	7608	2039	7.22
	60%	26390	7520	1919	7.26
Brain	8%	38047	12470	3267	8.59
	21%	37903	12994	3393	8.01
	60%	37954	10199	2908	8.54

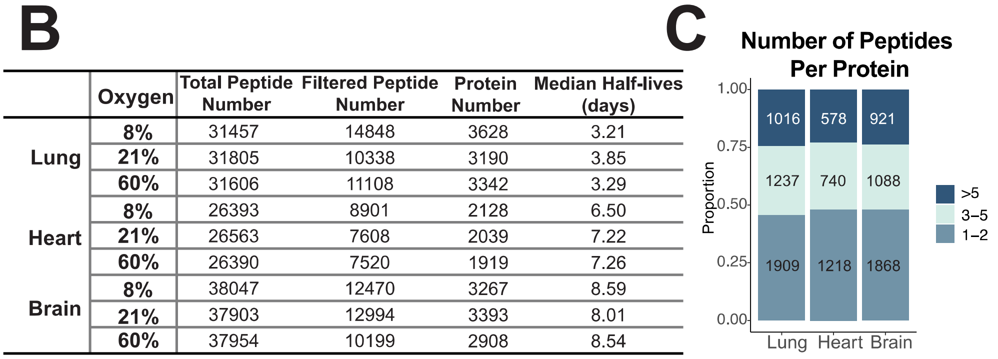

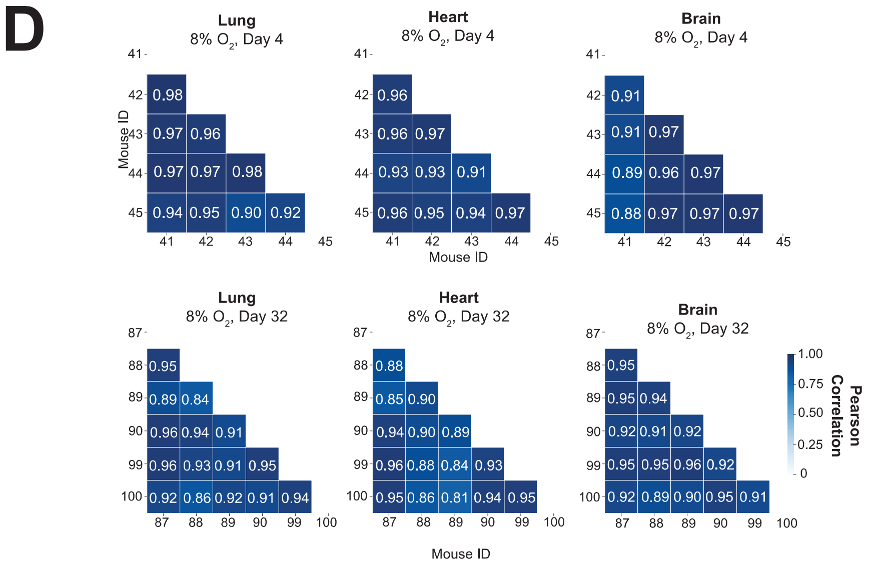

Figure S1. Analysis flowchart and quality control matrices of the pulsed-SILAM dataset.

A) Flowchart of the analysis pipeline. Peptides with only ^{14}N are quantified and normalized based on 10 previously identified long-lived proteins, under the assumption that the ^{14}N proportion of those proteins will stay constant over the course of the experiment. Then, the normalized data was divided by the abundance at time 0 to calculate the ^{14}N proportion for each time point. The preliminary fitting was performed using ordinary linear model for each peptide and the peptides were filtered based on the r^2 and the number of samples where they were detected. Next, the selected peptides were used for the estimation of protein degradation rates (K_d) using a mixed linear effects model, where peptides were set as random effects. To compare K_d between oxygen

tensions, a mixed linear effects model was applied, using oxygen and time as the fixed effects and peptides as the random variables.

B) Number of unique peptides detected in the mass spectrometry, number of unique proteins whose degradation rates estimated in the study, and median protein half-lives for each condition.

C) Number of peptides per protein used for protein degradation rate estimations.

D) Data reproducibility determined by the Pearson correlations between samples at different timepoints.

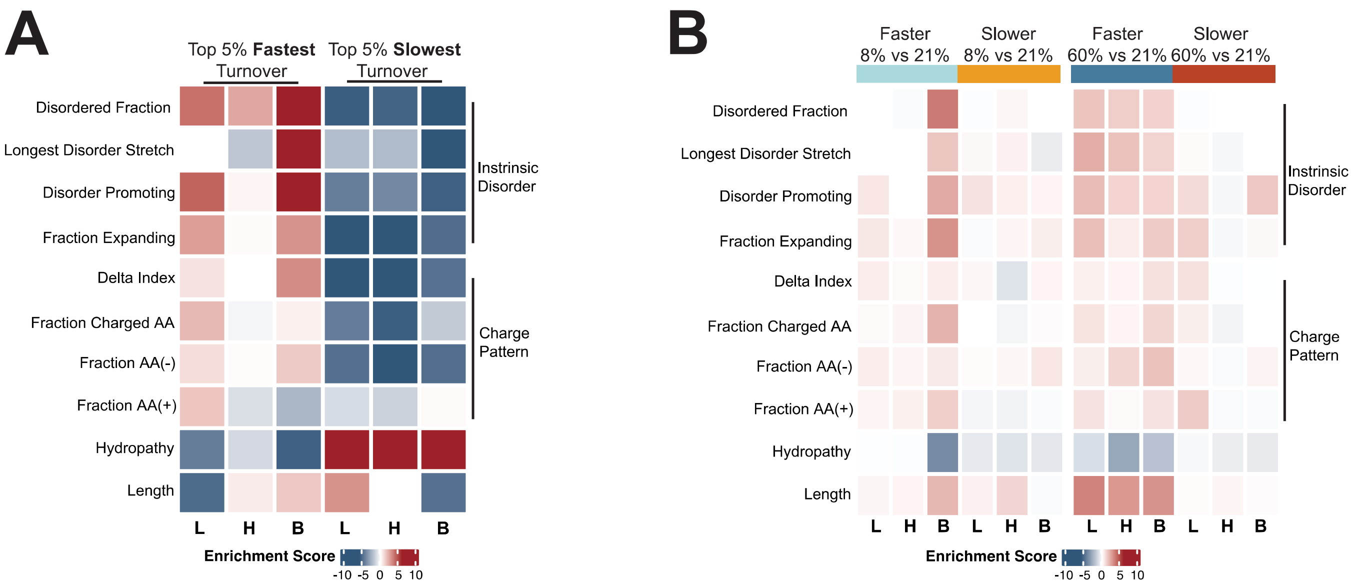

Figure S2. Protein half-lives are associated with biophysical features.

A, B) Enrichment of biophysical features for the proteins with the fastest or slowest degradation rates (A), or proteins with the largest oxygen-dependent changes in the degradation rates (B). “Disordered Fraction” and “Longest Disordered Stretch” were calculated using the DISOPRED3 method [70], where amino acids with scores above 0.5 are considered as disordered. "Disorder Promoting” was computed using the TOP-IDP-scale method [71]. "Fraction Expanding” refers to the fraction of residues that contribute to chain expansion (E/D/R/K/P) calculated using CIDER [72]. “Delta” score is a measurement for protein charge patterning calculated using the localCIDER algorithm [72]. A larger Delta score means that the charges are more unevenly distributed. “Fraction Charged AA”, “Fraction AA (-)”, and “Fraction AA (+)” refer to the fraction of charged residues, the fraction of negatively charged residues, and the fraction of positively charged residues, respectively. “Hydropathy” is the mean hydropathy as calculated from a skewed Kyte-Doolittle hydrophobicity scale [73]. “Length” refers to the protein amino acid length. The enrichment scores were calculated using the Kolmogorov-Smirnov test.

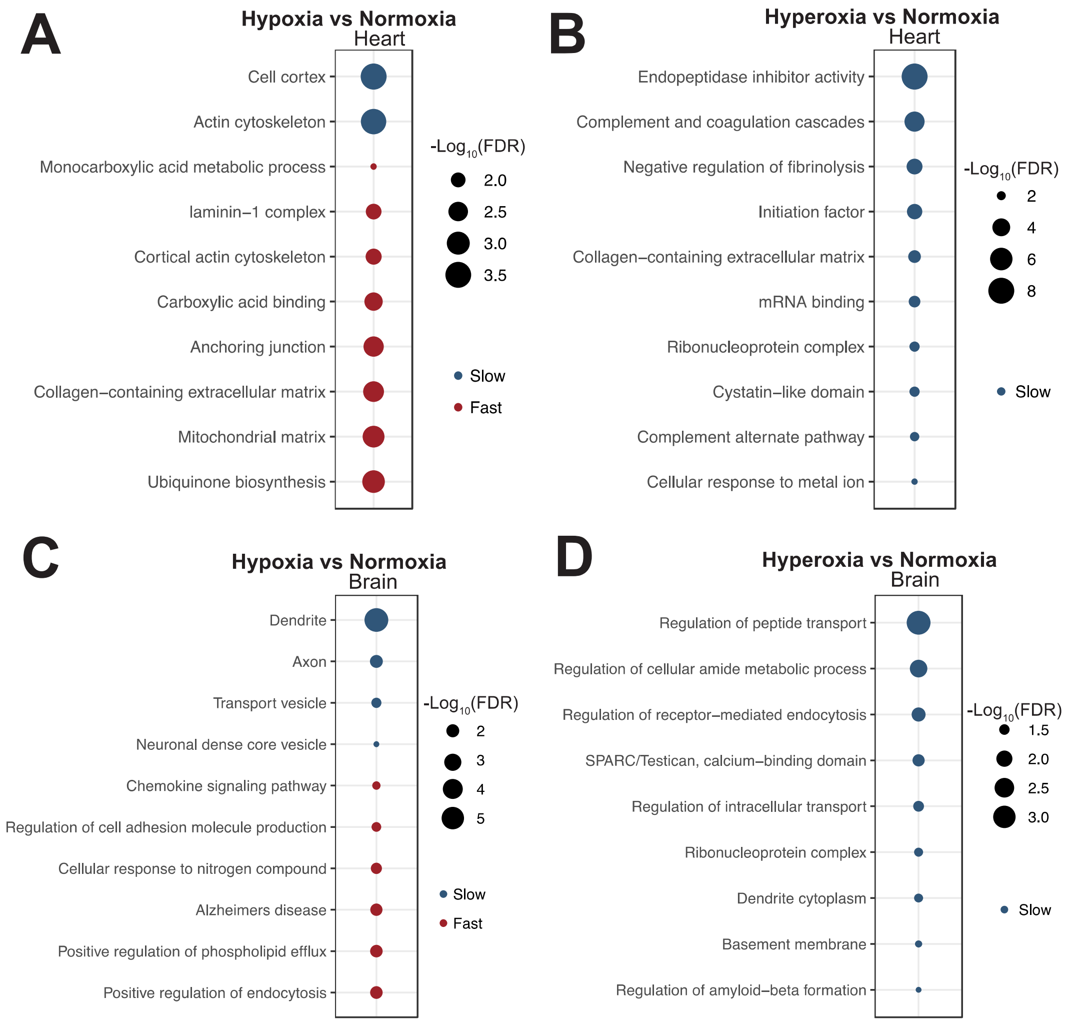

Figure S3. Oxygen-dependent protein turnover rates are tissue-specific.

A, B, C, D) Enrichment analysis for proteins with significant changes in protein degradation rates (FDR < 0.05, fold change > 1.3) in heart (A, B) and brain (C, D) tissues using the STRING functional analysis method. The size of the dots in represents the -$\log_{10}$(FDR) of each term.

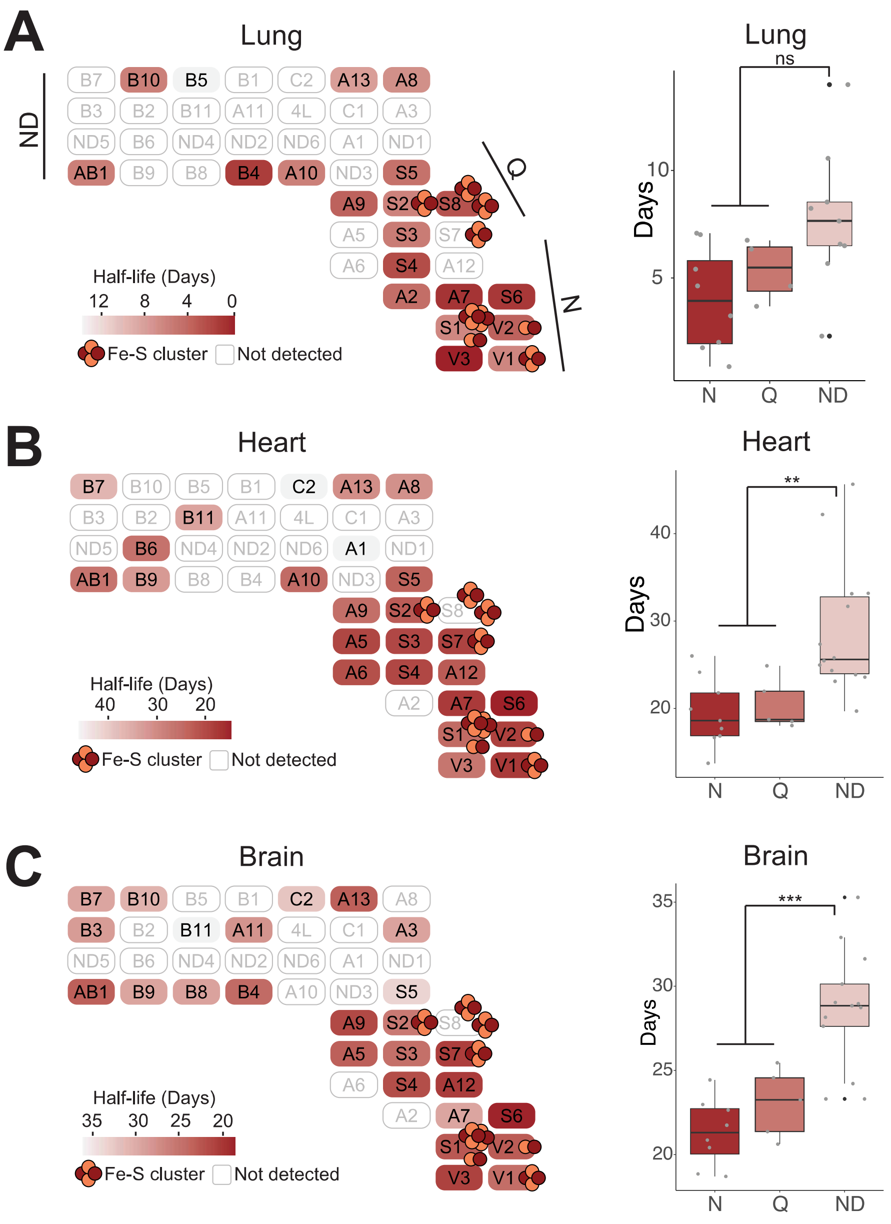

Figure S4. Different modules in Complex I of the electron transport chain (ETC) have different turnover rates.

A, B, C) Protein half-life in different subunits of ETC Complex I in normoxia in lung (A), heart (B) and brain (C). The box plots (right panels) show the half-life of proteins in the N-, Q- and ND-modules, where each dot represents the half-life of a subunit. The distributions of protein half-lives were compared between the subunits in the ND-module and those in the other two modules using the Kolmogorov-Smirnov test. ** $p < 0.01$, *** $p < 0.001$.

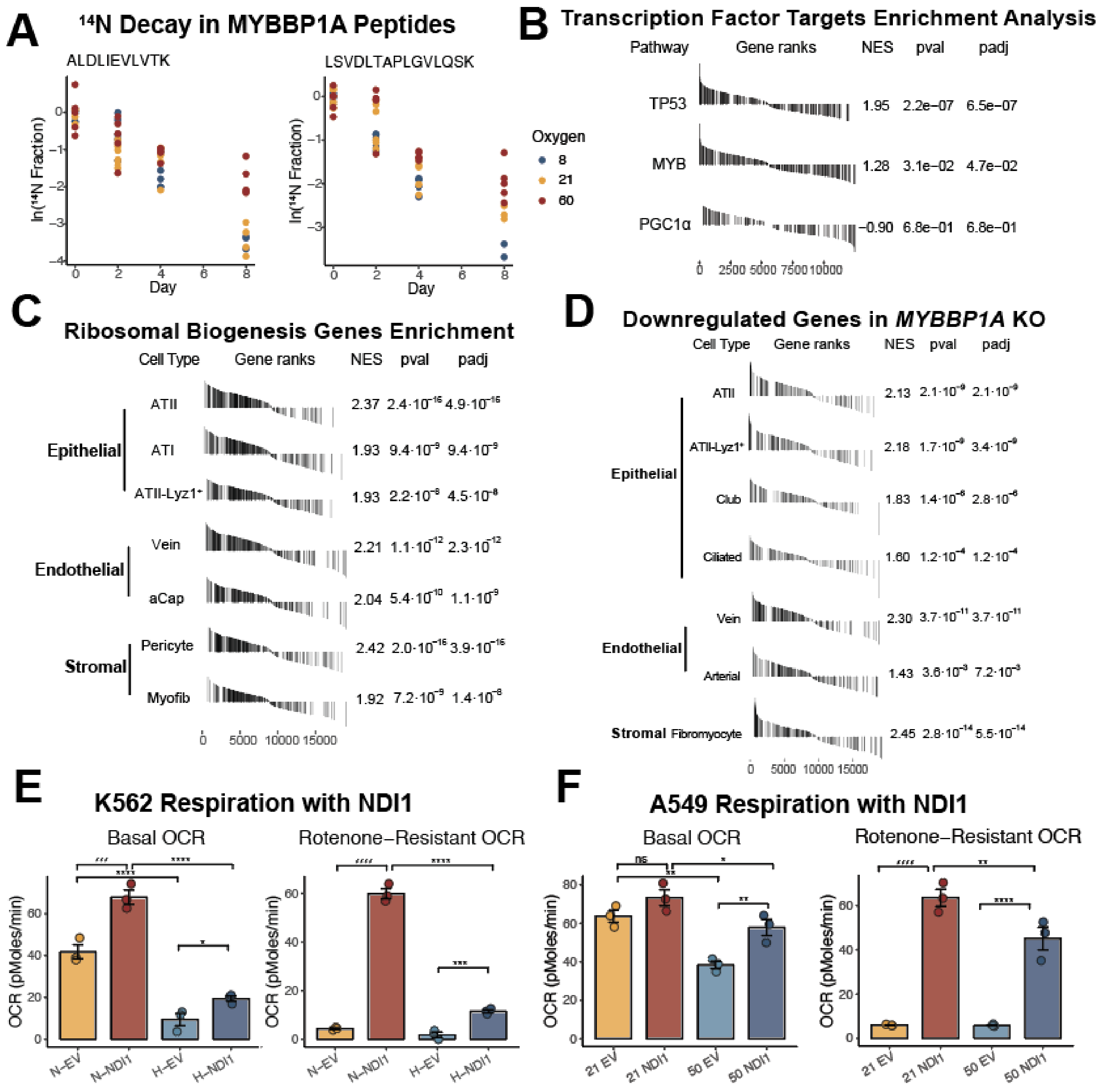

Figure S5. Enrichment analysis of transcription factor targets in the hyperoxic lung and effects of NADH/NAD ratio on mitochondrial respiration.

A) ^{14}N decay over time of 2 most abundance peptides of MYBBP1A in lung tissues. Each dot represents the abundance of each peptide in each subject.

B) Genes were ranked based on the fold changes in the QuantSeq dataset (from the most up-regulated to the most down-regulated in hyperoxia). The normalized enrichment scores (NES), and p values of the 3 transcription factor targets were calculated using the Gene Set Enrichment Analysis (GSEA) algorithm. The p values were adjusted using the Benjamini-Hochberg method.

C,D) Genes in each cell type were ranked based on the fold changes in the single-cell RNA seq data [45] (from the most up-regulated to the most down-regulated in hyperoxia). Ribosomal biogenesis genes **(C)** or the top 200 downregulated genes in *MYBBP1A* KO K562 cells (rank by Z-scores) **(D)** were analyzed using the GSEA algorithm in each lung cell type. The cell types with the most significant enrichment are shown.

E, F) Basal and rotenone-resistant mitochondrial oxygen consumption rate (OCR) in K562 with NDI1 or empty vector (EV) expression determined by Seahorse metabolic flux analysis after 4-day exposure in normoxia (N) or hyperoxia (H). Cells were sequentially injected with rotenone and antimycin to determine basal and rotenone-

resistant mitochondrial OCR. Each datapoint represents the average of 5 technical replicates. Mean +- SEM of biological triplicates is shown. Unpaired t-test, **** $p < 0.0001$, *** $p < 0.001$, ** $p < 0.01$, * $p < 0.05$.

SUPPLEMENTAL TABLE LEGENDS

Table S1 Long-lived proteins used for normalization.
Table S2 Degradation rates (K_d), half-lives and statistical analysis of the SILAM results in the 3 organs.
Table S3 Pathway enrichment analysis for the proteins with significant changes in hypoxia or hyperoxia.
Table S4 Protein complex analysis in lung tissues.
Table S5 Genes co-essential with MYBBP1A in the DepMap database.
Table S6 Differential RNA expression analysis in lung exposed to hyperoxia or normoxia.
Table S7 Pathway enrichment analysis for differentially expressed genes in hyperoxic lung.
Table S8 qPCR primers and antibodies used for cell isolation.
